# Endometriosis in later life: an intersectional analysis from the perspective of epistemic injustice

**DOI:** 10.1007/s11019-024-10245-4

**Published:** 2024-12-20

**Authors:** Elisabeth Langmann, Anna-Christina Kainradl, Merle Weßel, Alekszandra Rokvity

**Affiliations:** 1 Institute of Ethics and History of Health in Society, University of Augsburg, Universitätsstr. 2, 86156 Augsburg, Germany; 2 Center for Interdisciplinary Research on Aging and Care (CIRAC), University of Graz, Schubertstraße 23/I, Graz, 8010 Austria; 3 City of Hannover, Hannover, Germany

**Keywords:** Endometriosis, Menopause, Gendered ageism, Epistemic injustice, Bioethics

## Abstract

Endometriosis, a chronic inflammatory condition affecting 10% of biological women, is widely understudied and particularly overlooked in later life. Discussions surrounding endometriosis predominantly centre on medical gender bias during reproductive years, with limited attention to intersecting factors of discrimination and the impact of ageism on affected individuals. As endometriosis is framed as a disease of reproductive age, research is lacking when it comes to the effects of the illness on the older population. Symptoms in (post)menopausal individuals are frequently misattributed to other ailments due to ageist and sexist preconceptions, leading to prolonged diagnoses and mistreatment. This is a social justice issue in which age and sex contribute to the discrimination of a certain population – namely older biological women living with endometriosis. In this paper, we approach this issue from the perspective of epistemic justice. The experiences of the affected persons are shaped by a lack of knowledge about endometriosis among both the healthcare personal and the affected person, as well as a lack of acknowledgement and consideration of the persons experiences. Using the lens of epistemic justice, we develop an analytical model to understand the intersection of age and gender in the experiences of endometriosis patients. This article contributes to ongoing debates on epistemic injustice and intersectionality within medicine and healthcare, offering an analytical model that connects the critical approaches of epistemic injustice and intersectionality to address health injustice. Ultimately, this work advocates for a comprehensive, lifespan approach to endometriosis that acknowledges and addresses intersecting forms of discrimination.

## Introduction

Endometriosis, an estrogen-dependent inflammatory chronic condition characterized by the growth of tissue similar to the lining of the uterus outside the uterus, affects millions of people worldwide (Arafah et al. 2021). Despite the estimated prevalence of about 10% of biological women being affected (Moradi et al. 2021; Smolarz et al. 2021), the exact cause of endometriosis remains elusive. At the same time, the understanding and acknowledgment of this disease is influenced by misconceptions and disparities. Endometriosis is often associated with reproductive age and menstruation, leading to a lack of attention to its impact on other demographics. Studies have shown that a significant number of women continue to be affected by endometriosis well into their perimenopausal and postmenopausal years (Haas et al. 2012; Cope et al. 2020). The lack of recognition of endometriosis in older age is a stark manifestation of a broader phenomenon within the healthcare system.

In recent years, a growing body of research has highlighted the influence of sexism and ageism on the quality of healthcare, health outcomes, and access to care (Merone et al. 2022; Zhang et al. 2021; Schmitz and Lazarevič 2020). The intersection of two social categories may result in experience of inequality and discrimination, for example the invisibility of older biological women at all levels of healthcare (Krekula et al. 2018; Rochon et al. 2021). Looking at the intersecting dynamics of marginalization in relation to gender and age, a significant impact on older biological women becomes visible, which can pose a lasting threat to their health and well-being (Chrisler et al. 2016). Compared to other demographics, they are less likely to be taken seriously, receive poorer treatment, and have their emotional and psychological needs addressed less (Samulowitz et al. 2018; Macrae 2018). In this paper, we examine the intersection of age and gender in the diagnosis and treatment of endometriosis through the lens of epistemic injustice. Our primary research question investigates the extent to which the diagnostic and treatment challenges faced by older women with endometriosis are related to the epistemic injustices they experience in medicine and healthcare.

Epistemic injustice emerged as a concept that relates injustice to the production (and therefore recognition) of knowledge. Fricker (2007) defines epistemic injustice as the systematic neglect or discrediting of statements of members of certain groups. Epistemic injustice may occur in two different forms: as testimonial injustice – when the testimony of people due to their belonging to a group is not believed – or as hermeneutic injustice – when a lack of interpretative resources, shared understanding and knowledge hinders people who experience injustice to voice it (Fricker 2007). By examining endometriosis in women during later life, particularly (post)-menopause, we introduce new perspectives on how the intersection of age and gender contributes to social injustices in medicine and healthcare. We highlight significant analytical gaps in current endometriosis research, which predominantly focuses on the intersection of younger age (reproductive age) and gender. This is particularly important as many individuals diagnosed with endometriosis reach post-reproductive age and face unequal treatment and discrimination. Therefore, it is crucial to include this group of women in endometriosis research, not only to ensure they receive a good level of care and improve their quality of life, but also to uphold their fundamental right to health.

## Background

The establishment and growth of endometriotic tissue is estrogen-dependent and therefore mostly symptomatic in biological women of reproductive age, although endometriosis is present in the body before reproductive age and well into post menopause (Signorile et al. 2010; Becker et al. 2022). Symptoms of endometriosis can be chronic or cyclical. According to the European Society of Human Reproduction and Embryology (ESHRE) Endometriosis Guideline, the (British) National Institute for Health and Care Excellence (NICE), and the Endometriosis Foundation of America guidelines, the most prevalent symptoms of endometriosis are: abnormally severe uterine contractions during menstruation, abnormally heavy menstrual bleeding, painful sexual intercourse, chronic pelvic pain, ovulation pain, infertility, bowel and urinary disorders, chronic fatigue, cyclical cough and chest pain, and nerve pain in lower extremities (Becker et al. 2022; National Institute for Health and Care Excellence 2017; Endometriosis Foundation of America).

Although the vast majority of endometriosis research focuses on biological women in reproductive age, this heterogenic group faces stigmatization and challenges in obtaining adequate care (Tragantzopoulou 2024). The diagnostic delay of endometriosis is particularly problematic, as it is a “hallmark of a disease that can have at times crippling effects on individuals suffering from its associated symptoms and impact on their lives” (Becker et al. 2022). The reason behind this delay is understood to be influenced also by cultural conceptions and attitudes towards menstruation: those who menstruate are actively encouraged to conceal their menstruation, information and communication about pain as a menstrual symptom is lacking, and shame is an often-experienced feeling. Living with painful menstrual symptoms is connected to the concept of “normal” womanhood. “Prior to diagnosis, women experience repeat visits to doctors where their symptoms are normalized, dismissed or trivialized, leaving women feeling disbelieved or ignored” (Culley et al. 2013).

There is no known cure for endometriosis at this time. Surgical removal of endometriotic lesions and associated cysts and tissue is one treatment strategy; the other is the prescription of non-steroidal anti-inflammatory drugs and hormonal therapy to manage symptoms and suppress the hormonally active ectopic endometriotic tissue (Rafique and Decherney 2017). However, these treatment options have been tailored to patients of reproductive age, with the probability of adverse consequences in patients of older age not being taken into consideration.

### Confronting misconceptions: endometriosis and menopause

Despite the awareness that endometriosis can persist well into (post)menopause, research on this phase of the illness remains scarce. This scarcity is likely due to the long-standing myth that endometriosis only affects biological women of reproductive age and will vanish with the end of menstruation (Secosan et al. 2020). The effects of this myth are evident in national as well as international guidelines on menopause, which largely do not mention endometriosis. For instance, the European Menopause and Andropause Society (EMAS) published the position statement “*Managing the menopause in women with a past history of endometriosis”* in 2010, consisting of a literature overview and expert opinions (Moen et al. 2010). However, despite significant advancements in endometriosis research, the text has not been updated for over a decade. In 2022, EMAS did publish *Menopause*,* wellbeing and health: A care pathway from the European Menopause and Andropause Society*, but this paper does not mention endometriosis at all. Similarly, the *North American Menopause Society* does not offer any open access materials on the intersection of endometriosis and menopause (Lambrinoudaki et al. 2022). They mention endometriosis only twice on their website, both times very briefly: once in the glossary of terms, and once as a possible cause of painful sexual intercourse. Similarly, the recently updated NICE guidance on menopause does not address endometriosis (National Institute for Health and Care Excellence 2024). The Australasian Menopause Society (AMS) and the Canadian Menopause Society do mention endometriosis in guides that discuss hormonal therapy (Australasian Menopause Society; Canadian Menopause Society 2023). However, the guide created by the Canadian Menopause Society aims at health professionals and not the public.

The negligence of (post)menopause when it comes to endometriosis research, representation, awareness, diagnosis, and treatment lies also at the intersection of the cultural conceptions of both endometriosis and menopause, influencing biomedical discourses and interacting with societal inequality factors such as gender, age, race, and socio-economic background. Historically, menopause has been a topic of discourse since antiquity. Yet the understanding of the change from the reproductive to the non-reproductive phase of life of women changed over time and place (Poole 2023). Nevertheless, the nineteenth century saw with the publication of English physician Edward Tilt a first shift of pathologization of the menopause (van de Wiel 2014). A further significant shift in cultural and biomedical perspectives occurred with the discovery of the hormone estrogen by Edward Doisy and Adolph Butenaldt in 1929. This discovery led to the rebranding of menopause as “estrogen deficiency”, which resulted in its pathologization (Pickard 2022; Mattern 2021). The current medical definition of menopause according to WHO is the end of monthly menstruation due to loss of ovarian follicular function (World Health Organization 2022). Nevertheless, menopausal discourses in the Global North often associate menopause with much more than the end of menstruation; it is linked to loss of beauty, weakness, and declining health (Cruikshank 2013). Just as Gullette (1997) observes in describing the menopause playing “a powerful cultural role, in marking a (negative) shift in women’s social status, shaping both social norms and women’s self-appraisals and dividing women’s life course into two: fertile and post-fertile with value attributed only to the former” (Pickard 2022). The repercussions of this characterization are also evident in the (medical) evaluation of this stage of life, as Pickard reflects on her personal experience: “Dominant menopause discourses circulating in society today are far from being empowering. They treat this life stage as an illness or deficiency to be medicalised” (Pickard 2024).

### Misconceptions influencing endometriosis research

The drop of estrogen-production in menopause is associated with withdrawal of endometriosis symptoms, perpetuating the notion that with entering the menopause endometriosis symptoms will no longer occur (Gemmell et al. 2017). The standard treatment for menopause symptoms is hormonal replacement therapy (Gemmell et al. 2017). This is problematic for individuals with a history of endometriosis because hormone replacement therapy used to alleviate menopause symptoms can have two adverse effects: the influx of estrogen of external origin can “reactivate the growth of endometriosis deposits and cause symptom recurrence”, as well as “promote malignant transformation of residual endometriosis tissue” (Gemmell et al. 2017). In fact, recurrence of endometriosis lesions or the appearance of newly formed endometriosis in postmenopausal patients has been documented (Secosan et al. 2020). Yet, due to a lack of scientific studies, there are no clear guidelines, which leave healthcare providers without concrete recommendations (Secosan et al. 2020).

From a cultural perspective, it is relevant to observe that upon its discovery, estrogen started being seen as the essence of femininity (Mattern 2021). In the 1960s, physicians and pharmaceutical companies claimed that estrogen could preserve youth, attractiveness, and sexuality in older women “who would otherwise become dour, shrivelled shells of their former selves, destined to lose their husbands to younger women” (Mattern 2021). Considered a cure for menopause symptoms, estrogen therapy has been interpreted as a means of warding off old age. Cultural images and popular discourses on menopause intersect here. Being considered an old woman often corresponds to invisibility in popular representations as Cruikshank already noted in 2013: “[a] persistent problem is the near invisibility of older women’s health concerns in widely used texts.” (Cruikshank 2013). Even women in (post)menopause may internalize these ageist messages, leading to self-neglect and undervaluation of their own health needs. As Cruishank pointed out, “[j]ust as colonized people may internalize messages about their own inferiority […]” (Cruikshank 2013), (post)menopausal women may similarly undervalue their health concerns. This mindset is further perpetuated by the conspicuous absence of endometriosis in public and medical discourse surrounding menopause and can be associated with a lack of attention to older women’s health concerns and treatment options on a structural level. Furthermore, intersecting interpersonal dynamics shaped by sexism and ageism contribute to attitudes of healthcare professionals. The stereotyping of older biological women as asexual and in a state of physical and mental decline can foster a dismissal of endometriosis as a potential diagnosis, given that it is commonly associated with youthfulness and reproduction.

### Epistemic injustice and endometriosis in later life

Philosopher Miranda Fricker introduced the concept of epistemic injustice, distinguishing between “testimonial” and “hermeneutical” forms. Both types of injustice are related to the realm of knowledge and occur when someone is wronged in their “capacity as a knower” (Fricker 2007). Testimonial injustice arises when prejudice causes a listener to assign a lower level of credibility to a speaker’s word (Fricker 2007). For example, healthcare professionals may not believe a patient because of associations with their age or gender. In contrast, hermeneutical injustice stems from societal structures and norms that limit the interpretive resources available to marginalized groups. As such, this form of injustice occurs when there is a collective gap in interpretive resources, leaving someone at a disadvantage in understanding or explaining their social experiences. This can happen when society lacks the concepts or terms to describe certain experiences, placing the burden of interpretation on the affected individual. As an example, Fricker points to women experiencing postnatal depression before it was formally recognized as a medical condition (Fricker 2007).

Missing established frameworks are barriers for effectively communicating and interpreting feelings as well as experiences. Thus, applying the lens of epistemic injustice to the experience of endometriosis in later life means questioning established knowledge, common interpretations, and communication practices. It involves analyzing who is believed and whose experiences are dismissed, as well as analyzing the terms and concepts used to describe endometriosis. This approach helps revealing systemic issues in knowledge production and exchange, highlighting unequal representation and distorted communication. For instance, the historical association of female pain with hysteria or emotional instability can contribute to a dismissal of their symptoms as psychosomatic (Bourke 2014). Thereby, epistemic injustice has significant implications. It affects individuals’ ability to express their experiences and impacts which voices are valued or marginalized. Furthermore, it can manifest as exclusion, silencing, invisibility, or distorted representation, which may result in unjust differences in and credibility within communicative settings. Ultimately, epistemic injustice can undermine the status and agency of individuals in knowledge-based interactions (Kidd and Carel 2017).

### Testimonial injustice

Endometriosis and menopause in general present epistemic challenges in various ways with myths, stigma, and the normalization or disbelief of pain as central issues. Testimonial injustice is evident in this context, as endometriosis symptoms are often dismissed as “a bad period” or attributed to being “in one’s head” (Cole et al. 2021). Healthcare providers, friends and family members frequently meet those affected with scepticism and trivialization (Hawkey et al. 2022; Pettersson and Berterö 2020). As one person described feeling “absolutely crazy because no one believes that you’re in pain” in a study on experiences of women with endometriosis (Hawkey et al. 2022). This internalized disbelief exemplifies how societal attitudes contribute to undermining affected individuals’ credibility and confidence.

At the same time, endometriosis is connected to a challenging dynamic of knowledge and power between affected persons and healthcare professionals, including the perpetuation by clinicians of gendered stereotypes, inadequate knowledge of the disease among medical professionals, and neglect of female pain (Young et al. 2020). As a result, this often means that especially biological women of older age are not believed or taken seriously when they report symptoms, because endometriosis is understood as a disease of reproductive age. Besides, even when people are successfully diagnosed with endometriosis during their reproductive age, they are most often advised that menopause will mark the end of their illness. In practice, this means that in (post)-menopause the affected persons face additional prejudices and barriers when seeking treatment due to their age. Thereby this type of injustice is compounded by an intersection of sexism and ageism.

At the same time, older biological women are often perceived as less reliable sources of information about their own bodies (Cruikshank 2013). They face systemic disbelief and disregard of their menopausal symptoms from healthcare personal due to their intersectional status (Aririguzo et al. 2022). These misconceptions are compounded by intersectional factors such as race and socioeconomic status, further exacerbating the marginalization of older biological women (Okoro et al. 2022; Lu et al. 2022; Bougie et al. 2022). As one participant in a qualitative study exploring the experiences of African American women with healthcare noted: “Once again, just like the black women in our community lack knowledge, the practitioners in the healthcare field lack the knowledge on how to actually deal with African American women when it comes to, um – not only menopausal transition, but healthcare in general, and they tend to, um, give us the same fixes, for lack of a better word, or treatment” (Aririguzo et al. 2022). This highlights how bias can cause healthcare providers to dismiss or trivialize their reports of pain and other symptoms, attributing them rather to “normal” menopause or ageing than recognizing them as potential signs of a disease such as endometriosis (García-Rodríguez et al. 2023). Consequently, older biological women with endometriosis experience mistrust and skepticism, leading to prolonged suffering due to misdiagnosis, delayed diagnosis and treatment. Their voices are marginalized, and their experiences are invalidated, resulting in inadequate care due to the intersection of being female and older. This leads to their exclusion from epistemic participation and reflects both intrapersonal, interpersonal and structural biases. Furthermore, it may result in a potential lower quality of life due to mistrust in healthcare professionals as well as long term effects on physical and mental health.

### Hermeneutic injustice

Hermeneutic injustice refers to the ways in which cultural and societal biases can affect our understanding of a condition. Due to the significant lack of research and knowledge concerning the health and healthcare of ageing women (Kosiak et al. 2006; Krekula et al. 2018; Merone et al. 2022), biological women experience with hermeneutic injustice rooted in sexism and ageism in healthcare. Historically, on the one hand medical research has focused on biological men, resulting in a significant lack of knowledge and data on how diseases present and progress in individuals of other sexes. At the same time, female-specific research centers focus strongly on reproductive health, neglecting the needs of older women who are past menopause (Gilmer et al. 2023). On the other hand, medical knowledge is highly influenced by societal changes and trends. For example, doctors have reported that the rapid societal changes due demographic transformation, increasing visibility of diversity and migration left them with knowledge gaps. They face difficulties keeping up with these changes and the demands arising from them for their work (Lindenmeyer et al. 2016).

In the case of endometriosis in later life, hermeneutical injustice arises specifically from the lack of appropriate interpretive frameworks to understand the experiences of (post)menopausal women with endometriosis, along with insufficient recognition of the condition’s severity and impact on people’s lives. At the same time, the effects of endometriosis on overall health are only properly considered in 1% of studies on the biology of ageing, as highlighted in a recent study (Gilmer et al. 2023). This gross gap in research and thus knowledge represents a form of hermeneutic injustice, in which uniquely female health experiences are marginalized after reproductive age, leading to significant delays in diagnosing conditions such as endometriosis. The continuous medicalized gendered stereotypes surrounding biological women and their bodies contribute to fostering this lack of understanding. Limited medical research and understanding of endometriosis in this demographic contribute to a broader societal gap in recognizing and addressing these issues. This results in a misunderstanding of symptoms and inadequate public awareness, making it difficult for affected persons to find relevant information, care and support and thus situations of vulnerability.

The historical association of endometriosis with pathologies like hysteria have contributed to the disbelief and under-treatment of female pain symptoms (Guidone 2020; Hawkey et al. 2022). Similar accounts can be found regarding the female medicalised experience of menopause (Graham 2020). In turn, individuals may face being positioned as hysterical or difficult if they do not present themselves in certain ways in interactions with clinicians (Young et al. 2019). In this context, Kidd and Carel (2017) discuss the double burden of hermeneutical injustice often faced by people with chronic illnesses. Affected persons struggle to communicate the complex aspects of chronic illness, while simultaneously experiencing a sense of unspeakability, a feeling that they lack the precise language or means to express their experiences (Kidd and Carel 2017). This systemic bias not only perpetuates inadequate medical care but also silenced voices, preventing their experiences from being fully understood and addressed within the medical community, and as such, unjust denial of participation.

A notable manifestation of this hermeneutic injustice can be seen in the context of menopause and endometriosis. Symptoms might be mistakenly attributed to menopause instead of endometriosis, as healthcare professionals do often not recognize the interplay between these two conditions. For example, they do not understand the impact of hormone changes during menopause on endometriosis well. In particular, the strong focus on the importance of estrogen levels on the development of endometriosis, coupled with the assumption that decreased estrogen levels during menopause have a positive effect on endometriosis symptoms, can lead to other contributing factors being overlooked (Gemmell et al. 2017). These examples further illustrate the manifestations of hermeneutic injustice, where societal structures limit access to appropriate language and interpretive frameworks, thus creating a barrier to fair treatment with profound effects on overall well-being. By failing to adequately include endometriosis in menopause guidelines and resources as well as including older age in endometriosis guidelines, organizations such as EMAS or AMS, perpetuate a cycle of misinformation, wrongly guided perception, and neglect. This ultimately influences the quality of care and support available to the affected persons. As a result, healthcare professionals marginalize the experiences of (post)menopausal people with endometriosis due to insufficient interpretive resources. This marginalization is not merely a gap in medical knowledge but represents profound neglect to value and prioritize the health and well-being of (post)menopausal biological women and create unique health disparities and vulnerabilities.

## Discussion

The lack of menopause interpretative resources for both people living with endometriosis and healthcare providers, as well as the paucity of research and knowledge in this area, has left many people struggling with mistreated and misunderstood symptoms and complaints (Dillaway 2020; García-Rodríguez et al. 2023). The onset of menopause is marked by uncertainties in medical understanding and a lack of quality care, often resulting in poor quality of life during the transitional period, particularly for those experiencing conditions like endometriosis, as their symptoms are frequently neglected due to prevailing biases and knowledge gaps (Macpherson and Quinton 2022; Munn et al. 2022). Current medical research has disproven the assumption that endometriosis is exclusively a disease of the premenopausal period (Cope et al. 2020; Secosan et al. 2020; Dinu et al. 2024). In particular, due to the relatively high prevalence of the condition in affected persons aged over 40, the scarce available research recommends that physicians should consider endometriosis in cases of unclear pelvic pain in this age group (Haas et al. 2012).

At the same time, affected persons in these stages of life frequently encounter systemic biases that lead to their symptoms being trivialized or dismissed. Symptoms are not only misunderstood due to missing and incorrect information and research, reported symptoms are also frequently interpreted as less credible due to prevailing prejudices and stereotypes (Krekula et al. 2018; Rochon et al. 2021). As such, people in perimenopause and (post)menopause often experience increased probability of being exposed to epistemic injustice. Compounding the issue, there is evidence of symptoms being over-attributed to menopause when they may have different causes, such as endometriosis. Additionally, endometriosis is frequently confused with other conditions affecting the pelvic area (Hudson 2022). This adds significantly to the ongoing marginalization and inadequate treatment of individuals experiencing this condition in later life. Therefore, further research should systematically explore the intersection of age and gender to provide a more comprehensive understanding of the disease and the lived experience of illness as well as the associated injustice and discrimination faced by the affected persons. Employing participatory research methods, particularly with individuals from diverse backgrounds, is essential for ensuring research relevance and reducing hermeneutic injustice.

As long as misconceptions, misinformation, and mistreatment prevail, the symptoms and complaints of people with endometriosis beyond reproductive age will continue to be frequently overlooked and/or misinterpreted. While evidence highlights everyone living with endometriosis experiences injustice to a certain degree, our analysis shows that examining this phenomenon through the lens of epistemic injustice reveals additional dynamics of marginalization faced by specific groups. This underscores the critical need for a more nuanced understanding of the condition, especially compounded for those who also face other forms of stigma, i.e. belonging to marginalized social groups concerning race, class, disabilities, or other gender and sexual orientations. Specifically in the context of menopause, research has shown that further social categories have a significant impact and must be considered in further studies when analysing epistemic injustice in healthcare (e.g., (Bougie et al. 2022; Jones 2016; Kochersberger et al. 2024; Namazi et al. 2019). Thus, it is important to emphasize that endometriosis in later life is not a homogeneous phenomenon and to recognize the diversity of people living with endometriosis and their heterogeneous experiences.

Alongside the need for increased efforts to overcome gendered and ageist biases in medical research and practice, we recommend adopting a lifespan perspective on endometriosis that encompasses different stages of life and moves beyond rigid age categories, to capture the full spectrum of the disease. This approach should focus on addressing the specific needs of individual persons rather than limiting the analysis depending on age. This entails establishing research on the intersecting factors of discrimination, integrating the perspectives of people living with endometriosis at every age, updating guidelines to reflect the latest evidence, including the persistence of endometriosis beyond reproductive age, and providing clear protocols for the management of endometriosis in postmenopausal older individuals.

## Conclusion

Our analysis highlights that the interplay of age and gender significantly contributes to multifaceted forms of injustice in the context of endometriosis in later life. Fricker’s concept of epistemic justice offers a valuable tool to examine these diverse forms of injustice. This becomes particularly evident in the experiences of biological women in (post)menopause leaving with endometriosis. The widespread myths and misconceptions surrounding menstruation, menopause, and female reproductive health in general account for the societal lack of awareness, understanding, and recognition of endometriosis as a serious life-long chronic illness. Through our analysis, we show that this creates barriers for individuals to communicate their experiences of living with the disease and receiving validation. As such, we highlight that individual experiences are rendered invisible or are falsely categorized. The marginalization of the voices and experiences of people living with endometriosis in later life not only perpetuates a cycle of neglect and misinformation, but also reinforces significant barriers to exercising the right to health. Our analysis demonstrates that testimonial and hermeneutic injustice profoundly affect the lived experiences of those with endometriosis in later life. They are affected by systemic biases and unjust healthcare practices, resulting in inadequate recognition, misdiagnosis, and insufficient treatment options. Consequently, these factors reinforce entrenching inequalities in healthcare and undermine the epistemic capacity and agency of those affected. Thus, the lack of scientific research and comprehensive guidelines addressing endometriosis in (post)menopausal individuals highlights a critical gap. It also reflects a broader issue of gendered and ageist biases in medical research and healthcare, which perpetuates systemic inequities, marginalizes health concerns of older biological women, and undermines their capability to achieve well-being as they grow older. Thus, it is important not only to focus on the effects of marginalization but also on the underlying causes of health disparities. For future research on endometriosis, we therefore recommend an intersectional lifespan perspective. This includes the overall acknowledgment of the persistence of endometriosis beyond reproductive age but also researching intersecting discrimination factors, integrating participatory perspectives, updating guidelines to reflect current evidence, and providing clear management protocols for (post)menopausal individuals. By adopting this approach, we aim to pursue health equity and ensure that the healthcare needs of individuals with endometriosis are comprehensively addressed throughout their lives.

## Abbreviations

AMS: Australasian Menopause Society
EMAS: European Menopause and Andropause Society
ESHRE: European Society of Human Reproduction and Embryology
NICE: National Institute for Health and Care Excellence
